# Development of a Cardio-Oncology Service in Lithuania: Prediction, Prevention, Monitoring and Treatment of Cancer Treatment-Induced Cardiotoxicity

**DOI:** 10.3390/jcdd9050134

**Published:** 2022-04-26

**Authors:** Eglė Čiburienė, Sigita Aidietienė, Greta Ščerbickaitė, Birutė Brasiūnienė, Monika Drobnienė, Edita Baltruškevičienė, Tadas Žvirblis, Jelena Čelutkienė

**Affiliations:** 1 Clinic of Cardiac and Vascular Diseases, Institute of Clinical Medicine, Faculty of Medicine, Vilnius University, M.K. Čiurlionis Str. 21, 03101 Vilnius, Lithuania; sigita.aidietiene@santa.lt (S.A.); greta.scerbickaite@santa.lt (G.Š.); jelena.celutkiene@santa.lt (J.Č.); 2 Department of Medical Oncology, National Cancer Institute, 08406 Vilnius, Lithuania; birute.brasiuniene@nvi.lt (B.B.); monika.drobniene@nvi.lt (M.D.); edita.baltruskeviciene@nvi.lt (E.B.); 3 Clinic of Internal Diseases, Family Medicine and Oncology, Institute of Clinical Medicine, Faculty of Medicine, Vilnius University, 08661 Vilnius, Lithuania; 4 Department of Mechanical and Material Engineering, Vilnius Gediminas Technical University, 10223 Vilnius, Lithuania; tadas.zvirblis@gmail.com; 5 Institute of Biomedical Sciences, Faculty of Medicine, Vilnius University, M.K. Čiurlionio Str. 21, 03101 Vilnius, Lithuania

**Keywords:** cardio-oncology, cardio-oncology service, cancer, cardiotoxicity, survival

## Abstract

***Background***: Advances in cancer therapy have dramatically improved outcomes for cancer pa-tients. However, cancer treatment can cause several cardiovascular (CV) complications, increasing cardiac mortality and morbidity in cancer patients and survivors. As a result, a new cardiology subspecialty—cardio-oncology (CO)—has been developed. The goals of CO are to understand the mechanism of the cardiotoxicity (CTX) of cancer therapies and invent the best monitoring and treatment strategies to improve the survival of cancer patients. ***Methods***: We performed a retro-spective observational study reporting on the 6-year experience of the first CO service in Vilnius, Lithuania. Cancer patients were consulted by a single part-time specialist at Vilnius University Hospital. All new patients underwent blood tests, including cardiac biomarkers and advanced transthoracic echocardiogram (TTE) with stress protocol if indicated. During a follow-up, we evaluated the association of patient survival with such variables as age, gender, reasons for re-ferral, cancer location and stage, cardiovascular (CV) risk factors (RF), and rates and stage of CTX and treatment strategies. ***Results***: 447 patients were consulted (70% females), and the median age was 64 years. Cardiovascular (CV) RF was common: 38.5% of patients had hypertension, almost 38% had dyslipidemia, 29% were obese, 10% were smokers, and 9% had diabetes. Nearly 26% of patients had a history of HF. Early biochemical cardiotoxicity was determined in 27%, early functional cardiotoxicity was seen in 17%, and early mixed cardiotoxicity—in 45% of referred patients treated with cardiotoxic cancer therapies. In addition, reduced left ventricular ejection fraction (LVEF) was found in 7% of patients. Beta-blockers (BB) were administered to 61.1% of patients, while angiotensin-converting enzyme inhibitors/angiotensin receptor blockers (ACEI/ARB) to 54.1% of patients. In addition, 18.3% of patients received loop diuretics and almost 12% mineralocorticoid receptor antagonists (MRA), respectively. A total of 143 patients died during the 6-year follow-up period. The leading cause of death was primarily cancer (92.3%). Only in 5.6% of patients, cardiovascular complications were reported as the cause of death, and 2.1% of deaths were due to the COVID–19 infection. We found that age (HR 1.020 [95% CI: (1.005–1.036)] *p* = 0.009); LV diastolic dysfunction (HR 1.731 [95% CI: 1.115–2.689] *p* = 0.015; NYHA stage II (HR 2.016 [95% CI: 1.242–3.272] *p* = 0.005; NYHA stage III (HR 3.545 [95% CI: 1.948–6.450] *p* < 0.001; kidney dysfunction (HR 2.085 [95% CI: 1.377–3.159] *p* = 0.001; previous cancer (HR 2.004 [95% CI: 1.219–3.295] *p* = 0.006); tumor progression (HR 1.853 [95% CI: 1.217–2.823] *p* = 0.004) and lung cancer (HR 2.907 [95%CI: 1.826–4.627] *p* < 0.001) were statistically significantly associated with the increased risk of all-cause death. ***Conclusions***: CO is a rapidly growing subspecialty of cardiology that aims to remove cardiac disease as a barrier to effective cancer treatment by preventing and reversing cardiac damage caused by cancer therapies. Establishing a CO service requires a cardiologist with an interest in oncology. Continuous education, medical training, and clinical research are crucial to success. Age, previous cancer, tumor progression, kidney dysfunction, left ventricular diastolic dysfunction, and NYHA stages were associated with increased mortality.

## 1. Introduction

Cardio-oncology is a relatively new field of cardiology that evolved due to a growing number of patients presenting to cardiology services before, during, or after cancer treatment. Cardio-oncology focuses on detecting, monitoring, and treating cardiovascular disease as a side effect of cancer treatment, which can cause cardiac dysfunction, hypertension, ischemia, valvular and pericardial disease, thromboembolism, arrhythmias, and are the primary cause of morbidity and mortality in the oncological population [1].

Cardiovascular diseases and cancer account for most of the deaths in Lithuania, and they are the leading causes of death in Lithuania. It accounted for 52.7% of all deaths (approximately 16,000 people) in 2020. In the last 10 years, the CV morbidity rate has been unchanged while the mortality rate decreased from 915 to 733 per 100,000 people [2].

In Lithuania, 8168 cancer deaths (18.9% of all deaths) were reported in 2020, and 17,073 new cancer cases were detected. The cancer rate has increased by 33% in the last 10 years, from 563 to 750 per 100,000 people. The most frequent cancer in men is prostate cancer (25.9%), followed by lung cancer (12.8%) and colorectal cancer (10.8%), whereas in women, breast cancer is the most common (21%), followed by colorectal cancer (11.4%) and uterus cancer (9.5%) [2,3].

The rising cancer prevalence and interaction between cardiovascular disease and cancer have resulted in the need for cardio-oncology services in Lithuania. The first CO service was established in Vilnius University hospital Santaros Clinics to determine the cardiovascular risk in cancer patients, diagnose the early cardiovascular dysfunction during cancer treatment, and provide appropriate prevention and treatment for cardiotoxicity.

We hypothesized that early cardiotoxicity is a frequent finding in cancer patients receiving cardiotoxic therapies, but progression to HF is rare, whereas the risk of cardiotoxicity is associated with the cardiovascular risk profile; the aim of our study was to identify which risk factors have the most significant impact on the development of early cardiotoxicity and cancer patients’ survival.

## 2. Methods

We retrospectively studied patients referred to the cardio-oncology service at Vilnius University Hospital Santaros Clinics between December 2014 and December 2020.

Patients were referred by medical oncologists (74.8%), primary care (15.3%), and other cardiologists (9.9%). Common reasons for referrals to cardio-oncology clinic were (1) baseline cardiovascular risk assessment before cancer therapy; (2) assessment and treatment of left ventricular dysfunction and HF; (3) hypertension induced by cancer therapy; (4) chemotherapy-induced vasospasm; (5) direct cardiac complications of cancer (pericardial effusion and cardiac AL amyloidosis), (6) cancer-associated thrombosis, (7) arrhythmias, (8) QTc prolongation, and (9) evaluation of cardiac tumors.

Patients with 23 types of cancer were consulted. Rarely consulted cancer types (skin (5 patients), brain (3 patients), pharyngeal (4 patients), and sarcomas (6patients)) we grouped into “others”.

Eleven groups of anticancer treatment were administered to our patients: anthracycline (doxorubicin and epirubicin); anti-HER2 therapy (trastuzumab and pertuzumab); vascular endothelial growth factor inhibitors (pazopanib, sunitinib, bevacizumab, regorafenib, lenvatinib, ponatinib, and axitinib); multikinase inhibitors (nilotinib, crizotinib, dabrafenib, trametinib, dasatinib, gefitinib, and imatinib); fluoropyrimidine drugs (5-fluorouracil, capecitabine, and gemcitabine); alkylating agents (cyclophosphamide, temozolomide, carboplatin, cisplatin, ifosfamide, and oxaliplatin); antimicrotubule agents (docetaxel, paclitaxel, vinorelbine, and eribulin); proteasome inhibitors (bortezomib and carfilzomib); immune checkpoint inhibitors (nivolumab); monoclonal antibodies (cetuximab, rituximab, rovalpituzumab, panitumumab, and denosumab); hormonotherapy (tamoxifen, fulvestrant, goserelin, anastrozole, letrozole, exemestane, triptorelin, enzlutamide, and abiraterone).

For every patient, we performed an electrocardiogram (ECG), advanced transthoracic echocardiogram (TTE) to estimate left ventricular ejection fraction (LVEF), LV diastolic function, global longitudinal strain (GLS), valves assessment, right ventricular (RV) function and other heart function abnormalities.

LVEF was assessed by Simpson’s 2D and the drop in LVEF by >10% to a value < 50% from baseline value as well as 2D GLS reduction < −18% was considered chemotherapy-induced CTX [4].

Diastolic LV dysfunction was identified when half of the these variables were abnormal: septal e’ < 7 cm/s, lateral e’< 10 cm/s, average E/e’ ratio > 14, LA volume index > 34 mL/m^2^, and peak TR velocity > 2.8 m/s [5].

Stress protocol (exercise stress testing, myocardial single-photon emission computed tomography (SPECT), or dobutamine stress test) was performed if indicated.

Cardiovascular risk assessment was accomplished for every patient, and risk factors modification was suggested. Antihypertensive drugs, statins, antiplatelet agents, or anticoagulants were prescribed, and optimization of current HF treatment was made according to the latest guidelines. All patients were presented with the benefits of moderate physical activity during cancer treatment [6].

Additionally, we measured serum cardiac biomarkers, such as troponin and natriuretic peptides (brain natriuretic peptide [BNP] or N-terminal pro-brain natriuretic peptide [NT-proBNP]), which facilitate to diagnose cardiotoxicity early [7]. Acute HF is unlikely when NT-proBNP < 300 ng/L or BNP < 100 ng/L. Cut-offs for chronic HF are NT-proBNP < 125 ng/L or BNP < 35 ng/L [8].

According to our laboratory parameters, elevated troponin I concentration was >35 ng/L for men and >16 ng/L for women.

Since cardiovascular dysfunction can occur at any time of cancer treatment, it may take several visits. Based on baseline risk assessment, a follow-up plan with TTE and cardiac-specific biomarkers was made for each patient. According to recent guidelines, prior cardiotoxic cancer treatment, medical history of CVD, and CV RF were combined to inform the surveillance protocol [9].

High and moderate baseline cardiovascular risk patients were monitored more often than low-risk patients. Moreover, all patients were recommended to attend a follow-up visit 12 months after the last chemotherapy cycle.

In the case of cardiotoxicity, guideline-based HF treatment was administered, and follow-up visits were planned. Cardiotoxicity was diagnosed, classified, and managed according to Royal Brompton Hospital myocardial toxicity classes [10]. Patients who developed cardiotoxicity and were prescribed cardioprotective treatment were monitored every 1–3 months until normalized test results.

Information about the reason and date of death was obtained from the Lithuanian Cancer Registry, a member of the International Association of Cancer Registries and European Network of Cancer registries. Lithuanian Cancer Registry periodically performs data linkage with the Lithuanian Causes of Death Registry, achieving data about the date and foremost reason of death and other information from the death certificate.

## 3. Statistical Analysis

Quantitative continuous variables are presented as minimal (min), mean, maximal values (max), and standard deviation (SD). For categorical variables, frequencies and proportions (percentages) of each category or combination of categories are presented. An independent sample t-test was used to compare the values of means between the two groups. One-way ANOVA was performed to identify the significant differences between more than two groups. The Chi-square test was used to evaluate the differences between two independent categorical data groups. A univariate Cox regression model was used to evaluate potential risk factors for overall survival. Factors found to be below 0.05 statistical significance level in univariate Cox regression analysis were entered into a multivariate model with a forward model selection process. Overall survival was defined as the time from the patient’s first visit to the cardio-oncology clinic to death from any cause. Data were obtained from the Lithuanian Cancer Registry. A two-tailed *p*-value < 0.05 considered being significant. Statistical analysis was performed using Statistical Analysis System (SAS) package version 9.2.

## 4. Results

We retrospectively studied 447 patients (70% females) referred to our service in 6 years between December 2014 and December 2020. The median follow-up was 18.5 months. The average visit frequency was 3.1 visits per person (1–14). The median age of the patients was 64, ranging from 18–92 years.

Medical oncologists referred most patients, and the most common reasons for referral were cancer treatment complications and pre-chemo/pre-operation risk assessment (45% and 42% of patients, respectively). The stress test was performed on 64% of patients.

**Figure 1:**
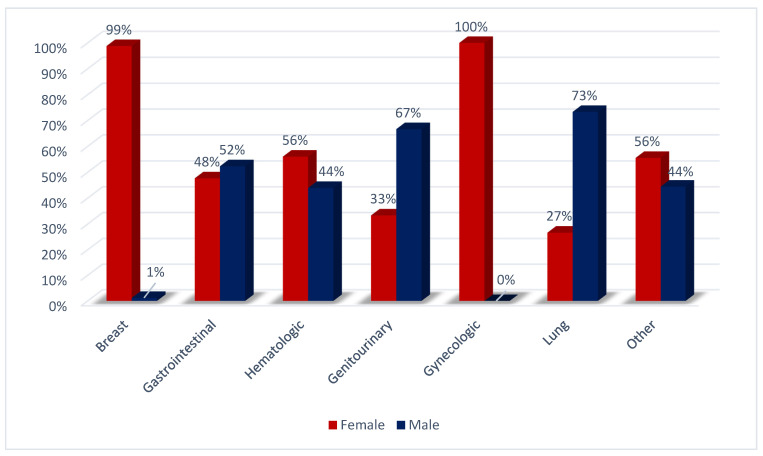
Sex differences in cancer incidence.

Patients with 23 cancer types were referred; more than half of the patients had an advanced cancer stage. The baseline characteristics of the patients are listed in Table 1. Cancer types’ gender distribution is shown in Figure 1.

### 4.1. Baseline Cardiovascular Risk Stratification in Cancer Patients and Their Personalized Surveillance Plan during Cardiotoxic Treatment

**Figure 2:**
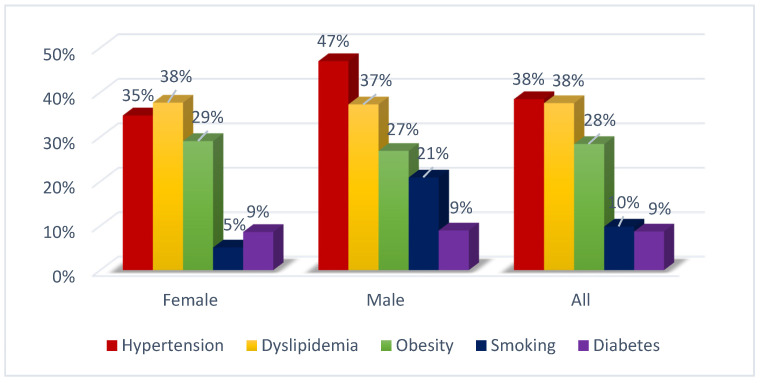
Cardiovascular risk factors in cancer patients.

CV risk factors were common among cancer patients (Figure 2). Therefore, to identify patients at increased risk for cardiotoxicity, careful baseline assessment of cardiovascular risk factors and prior cardiovascular diseases or prior exposition to cardiotoxic treatments are needed.

**Figure 3:**
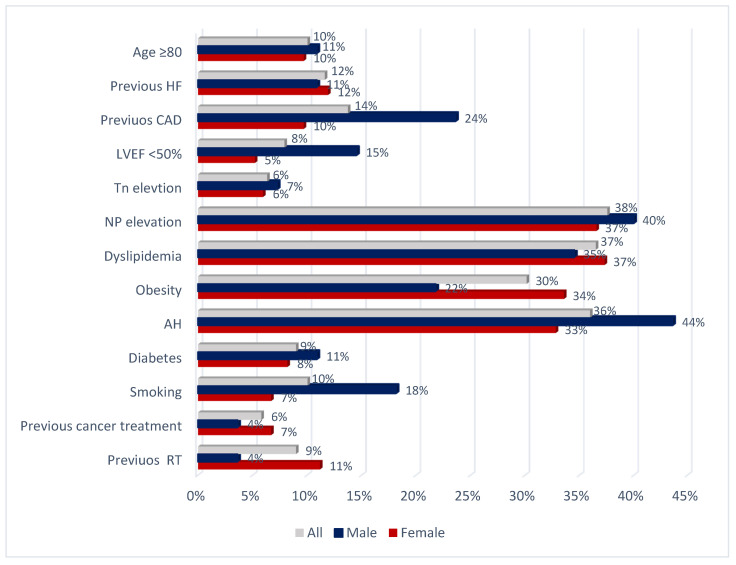
Cardiovascular cardiotoxicity risk factors in cancer patients prior to cardiotoxic cancer therapies. AH—arterial hypertension; CAD—coronary artery disease; HF—heart failure; LVEF—left ventricular ejection fraction; NP—natriuretic peptides; RT—radiotherapy; Tn—troponin.

One-third of 198 patients referred prior to cancer treatment were considered at a high and very high baseline risk. Main cardiovascular cardiotoxicity risk factors are presented in Figure 3.

Previous CVD and modifiable CV risk factors treatment was optimized for high baseline cardiovascular risk patients, and a personalized surveillance plan was recommended: cardiac biomarkers test and echocardiogram every two cycles of cardiotoxic chemotherapy. Medium-risk patients were suggested to consult a cardiologist at the end of cardiotoxic treatment or if any cardiac signs or symptoms appear. Low-risk patients should consult a cardiologist if any cardiac signs and symptoms manifest or 12 months after cardiotoxic treatment [11]. It took an average of 2–3 visits for low-risk patients, 3–5 visits for medium-risk patients and 4–7 for high-risk patients. Even more visits were needed after CTX was diagnosed.

**Figure 4:**
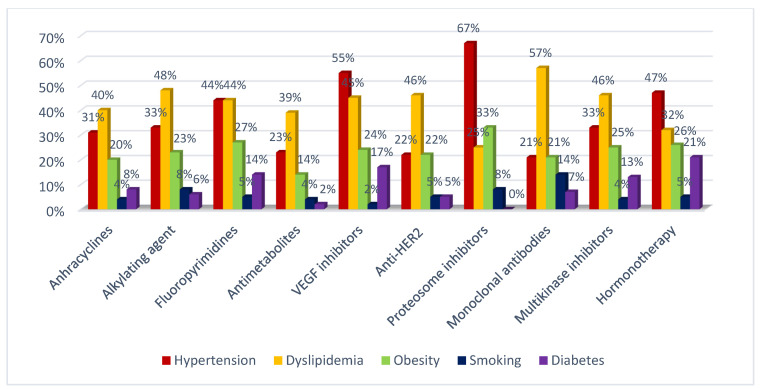
The relationship between anticancer therapies and cardiac risk factors.

The relationship of anticancer therapies and cardiac risk factors is shown in Figure 4.

### 4.2. Cancer Therapy-Induced Cardiotoxicity

45% of patients were referred for cardiac problems during cancer therapy. Symptomatic LVSD was determined in 13 (6.4%), while asymptomatic LVSD was seen in only one (0.6%) patient. Troponin elevation was found in 23 (11.3%), NP elevation in 50 (24.6%), GLS reduction in 17 (8.4%) and diastolic dysfunction in 24 (11.8%) patients.

**Figure 5:**
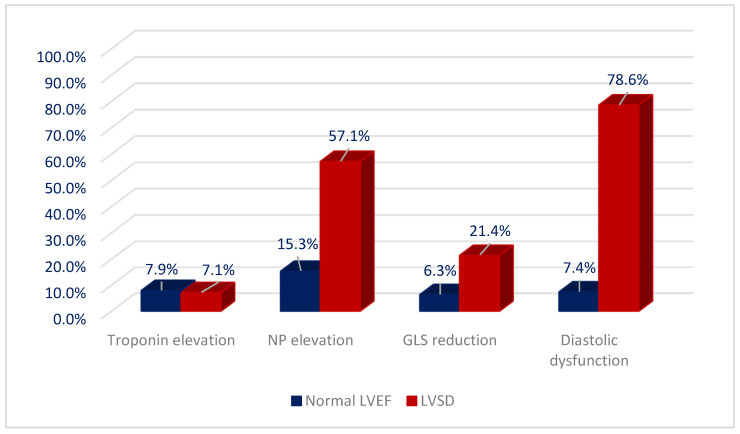
Myocardial damage markers in patients with normal and reduced LVEF. NP—natriuretic peptides; GLS—global longitudinal strain; LVEF—left ventricular ejection fraction, LVSD—left ventricular systolic dysfunction.

Early biochemical cardiotoxicity was determined in 44 (21.7%) patients, and early functional cardiotoxicity was diagnosed in 23 (11.3%) patients. In seven (3.4%) patients, we determined early mixed cardiotoxicity. Myocardial damage markers in patients undergoing cancer treatment are shown in Figure 5.

Any new troponin elevation above the upper limit of normal was considered subclinical cardiotoxicity, and cardioprotective treatment with ACEI/ARB or BAB was prescribed. These patients were re-consulted every 4–6 weeks, and if troponin continued to rise, BAB or ACEI/ARB was added at maximally tolerated doses. At every visit, an echocardiogram was performed to evaluate LVEF, diastolic function, and GLS.

Isolated NP elevation led to an increase in monitoring frequency.

New GLS reduction < −18% was acknowledged as early subclinical cardiotoxicity, and re-consultation after 4 weeks was administered. If GLS continued to decline, cardioprotective treatment was initiated.

### 4.3. Treatment Options

**Figure 6:**
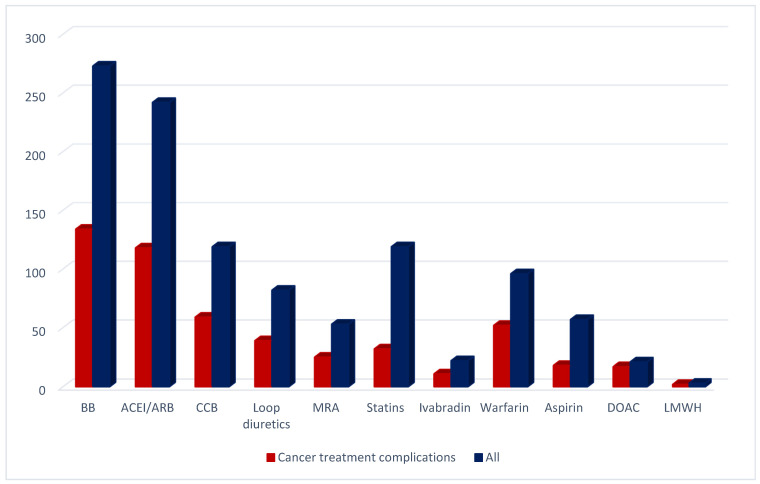
Treatment options. ACEI/ARB—angiotensin-converting enzyme inhibitor/angiotensin receptor blocker; BB—beta-blocker; CCB—calcium channel blocker; DOAC—direct oral anticoagulant; LMWH—low molecular weight heparin; MRA—mineralocorticoid receptor antagonist.

9.8% of patients have been prescribed contemporary HF treatment with ACEI/ARB, BAB, MRA and diuretics. Other treatment modalities are presented in Figure 6. Our patients received BAB more frequently than ACEI/ARB (53% vs. 47%).

When cardioprotective treatment was administered in case of troponin elevation, the normalization of troponin concentrations was observed after 4–8 weeks. Positive effects of HF were noticed after 6–8 weeks.

### 4.4. Impact of Prognostic Factors on Survival in Cancer Patients Referred to CO Clinic

During the study period, 143 (32%) patients died, more women than men.

**Figure 7:**
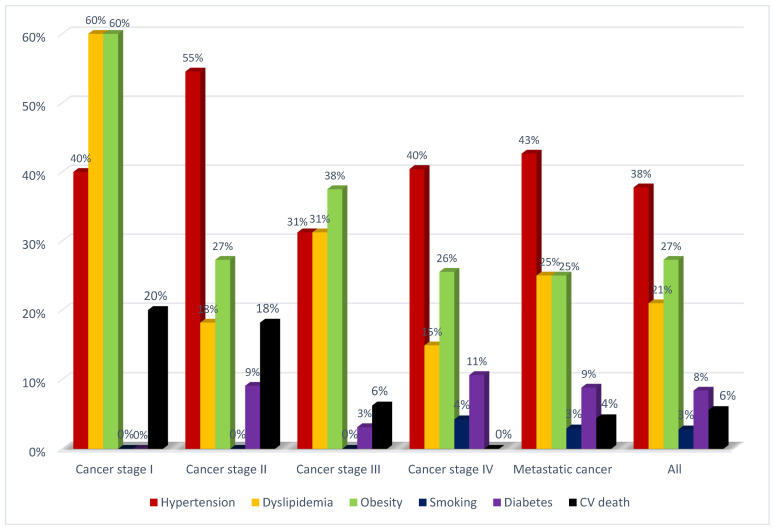
The cardiovascular risk profile and cardiovascular death rates of patients with different cancer stages. CV—cardiovascular.

Deceased patients were older and more frequently had elevated NP, LV diastolic dysfunction, and decreased GLS. In addition, these patients had more advanced cancer and HF NYHA stages, metastatic cancer, kidney dysfunction, cancer-associated inflammation, previous cancer history, and tumor progression (Table 2). The cardiovascular risk profile and rates of cardiovascular death of patients with different cancer stages are presented in Figure 7.

The prognostic impact of different factors is presented in Table 3. In univariate analysis, we found that age, NP elevation, LV diastolic dysfunction, decreased GLS, cancer stage III and IV, HF NYHA stage II and III, kidney dysfunction, CRP elevation, anemia, previous cancer, tumor progression and genitourinary, gynecologic and lung cancer were statistically significantly associated with increased risk of all-cause death. HF NYHA stage I and IV were excluded from analysis due to insufficient data.

Multivariable Cox regression analysis revealed that age, LV diastolic dysfunction, lung cancer, metastatic disease, NYHA stage II, III, kidney dysfunction, previous cancer, and cancer progression were the independent predictors of death (cancer stage, CRP, and GLS were excluded from the analysis due to insufficient data) (Table 4).

## 5. Discussion

Cardio-oncology service aims to improve the standard of care for oncology patients and cancer survivors treated with cardiotoxic cancer therapies or radiotherapy. New cancer therapies are being developed rapidly, extending cancer patients’ lives. However, this benefit comes with adverse cardiovascular effects. Therefore, comprehending the mechanism of adverse effects causing CTX and developing personalized treatment and follow-up strategies based on the most recent guidelines is mandatory in CO care.

Cardio-oncology services are relatively new, and data about their activities and results are limited. We presented the information about our CO service prediction, prevention, monitoring, and treatment of cancer treatment-induced cardiotoxicity strategy. We think that it is important to share experience in the phase of establishing CO service globally.

These are the first data showing an independent association between LV diastolic dysfunction and all-cause mortality in cancer patients. Cardiovascular risk factors (AH, diabetes, dyslipidemia, smoking, and previous cardiac disease) did not show predictive value for death in the population referred to our CO service. The rates of hypertension and diabetes were similar to other studied cancer patients, but rates of dyslipidemia and obesity were higher [10,12].

It is being studied what the most effective cardioprotective treatment in cancer patients undergoing cardiotoxic treatment is [13,14,15]. Data from the multiple small trials suggest that ACEI/ARB and BAB have a potential role in cardiotoxicity prevention and treatment. However, what is the most effective therapy is still unknown because of the lack of solid evidence. We prescribe BAB slightly more frequently than in other cardio-oncology services [12].

We consult cancer patients according to the latest cardio-oncology guidelines, research, and recommendations. Furthermore, continued education is needed to keep up with innovations.

Our results revealed that mild cardiotoxicity (abnormal biomarkers, some LV function abnormalities, and LVEF ≥ 50%) was diagnosed in 34.5% of patients, and this was consistent with results from the Cardiotox Registry data where mild cardiotoxicity was observed in 31.6% of cancer patients [12]. However, in the cardio-oncology service of Royal Brompton hospital, these rates were higher probably because they consult exclusively high baseline risk patients [10].

With age, the number of cardiovascular risk factors increases, their treatment is less intensive, and cardioprotective properties are diminished, leading to increased cardiotoxicity rates in the elderly [16]. A more advanced cancer stage may be associated with more intensive and/or complex cancer treatment, which can cause more cardiovascular complications. Anemia and renal insufficiency complicate not only cancer but also heart condition. It has already been proved that age, cancer stage, metastatic cancer, anemia, renal insufficiency, inflammatory status, and lung cancer is associated with increased mortality. However, our data showed that genitourinary and gynecologic cancer statistically significantly increases the risk of death.

Cardiovascular system assessment prior to cardiotoxic cancer treatment would help manage pre-existing CVD and modifiable CV risk factors. It would reduce the risk of CV complications during cancer treatment and decrease cancer treatment interruptions, but only a few patients receive this. Only a few referred patients had an echocardiogram, and no cardio-specific biomarkers or GLS were performed before cancer therapy started. Most patients are referred to a cardiologist only when the cancer therapy-induced cardiotoxicity occurs, although this could have been avoided if baseline cardiovascular risk had been assessed and the appropriate monitoring plan had been scheduled.

## 6. Study Limitations

These data represent a clinical experience from a single center and have limitations related to the retrospective nature of the study’s design: missing data on potential confounding factors. In addition, LVEF and GLS measurements are always influenced by the image quality and inter-vendor variability.

Another problem is with the cause of death coding in mortality statistics in our country: most often cause of death of cancer patients is coded as “cancer”, and information about underlying conditions is not available.

Most of the patients were referred to our center late when HF symptoms appeared, and no prior biomarkers or echocardiography were performed.

Dyslipidemia was diagnosed according to elevated total cholesterol and LDL cholesterol levels regardless of low HDL cholesterol level, which is also a cardiovascular risk factor.

## 7. Conclusions

CO is a rapidly growing subspecialty of cardiology that aims to remove cardiac disease as a barrier to effective cancer treatment and prevent, reduce, and reverse cardiac damage of cancer therapies. This would be impossible without mutual cooperation among oncologists and cardiologists. At least one cardiologist interested in oncology is needed to establish a CO service. Continuous education, medical training, and clinical research are crucial to success. It is vital to assess baseline cardiovascular risk in patients prior to cardiotoxic cancer treatment, and an appropriate monitoring plan should be scheduled. Moreover, biomarkers must be performed more often to diagnose cardiotoxicity early and start cardioprotective treatment to prevent HF. Early diagnosis and appropriate treatment of mild cardiotoxicity will help prevent cancer treatment interruptions and reduce the mortality of cancer patients.

## 8. Declarations

Consent for publication: The manuscript does not contain any individual person’s data in any form. All authors consent for publication.

## Figures and Tables

**Table 1:** Patient baseline characteristics.

Baseline Characteristics	All
	*n = 447* (%)
Age, years, (mean ± SD, range)	63.9 ± 18.3, 18–92
Female sex, *n* (%)	313 (70)
**Type of visit, *n* (%)**	
Pre-surgery/pre-chemotherapy	189 (42.3)
Cancer treatment complications:	203 (45.4)
LVD	14 (6.9)
hypertension induced by cancer therapy	79 (38.9)
Chemotherapy-induced vasospasm	16 (7.9)
Pericardial effusion	9 (4.4)
Cardiac AL amyloidosis	3 (1.5)
Cancer-associated thrombosis	17 (8.4)
Arrhythmias	48 (23.6)
QTc prolongation	13 (6.4)
Cardiac tumors	4 (2)
Post-treatment complications	55 (12.3)
**Cancer location, *n* (%)**	447 (100)
Breast	168 (37.6)
Gastrointestinal	86 (19.2)
Hematologic	66 (14.8)
Genitourinary	42 (9.4)
Gynecologic	37 (8.2)
Lung	30 (6.7)
Other	18 (4)
**Cancer stage, *n* (%)**	241 (53.9)
I	29 (12)
II	77 (31.9)
III	76 (31.5)
IV	58 (24)
Metastatic cancer	114 (25.5)
**CV risk factors, *n* (%)**	447 (100)
Hypertension	172 (38.5)
Diabetes	39 (8.7)
Dyslipidemia ^a^	168 (37.6)
Smoking	44 (9.8)
Obesity	127 (28.4)
History of HF	47 (10.5)
Prior CAD	42 (9.4)
Valvular heart disease	19 (4.2)
Kidney dysfunction (GFR < 60 mL/min/1.73 m^2^)	62 (17.1)
**Sinus rhythm, *n* (%)**	396 (88.6)
**Anticancer therapy, *n***	258 (57.7)
Anthracycline	75 (29.1)
Alkylating agents	84 (32.6)
Fluoropyrimidines	66 (25.6)
Antimetabolites	57 (22.1)
VEGF inhibitors	42 (16.3)
Anti-HER2 therapy	37 (14.3)
Hormonotherapy	19 (7.4)
Proteasome inhibitors	12 (4.6)
Monoclonal antibodies	14 (5.4)
Multikinase inhibitors	24 (9.3)
ICI	1 (0.4)
**Myocardial damage markers, *n* (%)**	
Tn I measured	278 (62.2)
Tn I elevation	32 (11.5)
BNP measured	329 (73.6)
BNP > 100 ng/L	112 (34)
BNP > 35 ng/L	221 (67.2)
NT-pro BNP measured	51 (11.4)
NT pro BNP > 125 if age < 75; > 450 if age > 75 years	17 (33.3)
LVEF < 50%	33 (7.4)
LVEF ≥ 40 and < 50%	21 (4.7)
LVEF < 40%	12 (2.7)
LAVI > 34 mL/m^2^	250 (70)
E/É ≥ 14	46 (12.3)
GLS < −18%	38 (23.5)
TAPSE ^b^ < 17 mm	17 (10.2)
S’ ^c^ < 12 m/s	3 (1.6)
**Previous cancer, *n* (%)**	31 (6.9)
**Previous chemotherapy, *n* (%)**	46 (10.3)
**Previous radiotherapy, *n* (%)**	41 (9.2)
**Cancer progression, *n* (%)**	88 (19.7)

BNP—brain natriuretic peptide; CAD—coronary artery disease; GFR—glomerular filtration rate; GLS—global longitudinal strain; HF—heart failure; ICI—immune checkpoint inhibitors; LAVI—left atrial volume index; LVD—left ventricular dysfunction; LVEF—left ventricular ejection fraction; NP—natriuretic peptide; NT—proBNP—N-terminal pro-brain natriuretic peptide; S’—tricuspid annular systolic velocity by tissue Doppler; SD—standard deviation; TAPSE—tricuspid annular plane systolic excursion, Tn I—troponin I; VEGF—vascular endothelial growth factor; VTE—venous thromboembolism. ^a^ Dyslipidemia was diagnosed when the total cholesterol level was >5.2 mmol/L, LDL cholesterol level >3 mmol/L. ^b^ TAPSE was measured in 166 patients. ^c^ S’ was measured in 192 patients.

**Table 2:** Deceased patients’ characteristics.

Patients Characteristics	Deceased Patients *n* = *143* (%)	Alive Patients *n* = *304* (%)	*p*-Value
Age, years, (mean ± SD, range)	67.6 ± 10.46, 33–92	62.2 ± 13.29, 18–92	**<0.001**
Female sex, *n* (%)	90 (62.9)	223 (73.4)	**0.025**
**Myocardial damage markers, *n* (%)** Troponin elevation NP elevation Left ventricular systolic dysfunction Left ventricular diastolic dysfunction Abnormal GLS	11 (14.5) 56 (48.3) 14 (9.8) 85 (69.7) 15 (42.9)	22 (10.8) 73 (29.4) 19 (6.3) 114 (42.4) 23 (18.1)	0.402 **<0.001** 0.182 **<0.001** **0.002**
Cancer location *, *n* (%) Breast Gastrointestinal Hematologic Genitourinary Gynecologic Lung Other	22 (15.4) 35 (24.5) 14 (9.8) 21 (14.7) 21(14.7) 23 (16.1) 7 (4.9)	158 (52.0) 51 (16.8) 52 (17.1) 21 (6.9) 16 (5.3) 7 (2.3) 11 (3.6)	**<0.001** 0.054 **0.042** **0.009** **<0.001** **<0.001** 0.522
**Cancer stage, *n* (%)** I II III IV	5 (5.3) 11 (11.6) 32 (33.7) 47 (49.5)	26 (14.6) 72 (40.4) 52 (29.2) 28 (15.7)	**<0.001**
**Metastatic cancer, *n* (%)**	68 (47.6)	47 (15.5)	**<0.001**
**HF NYHA stage, *n* (%)** 0 I II III IV	24 (17.6) 12 (8.8) 73 (53.7) 26 (19.1) 1 (0.7)	122 (46.2) 23 (8.7) 98 (37.1) 21 (8.0) 0	**<0.001**
**CV risk factors, *n* (%)** Hypertension Diabetes Dyslipidemia Smoking Obesity Prior CAD Valvular Heart Disease Kidney dysfunction (GFR < 60 mL/min/1.73 m^2^)	54 (37.8) 12 (8.4) 30 (21) 4 (30.8) 39 (27.3) 15 (10.5) 7 (4.9) 28 (19.6)	117 (38.5) 27 (8.9)138 (45.4) 16 (37.2) 88 (28.9) 28 (9.2) 12 (3.9) 34 (11.2)	0.883 0.864 **<0.001** 0.671 0.714 0.669 0.643 **0.017**
**CRP elevation, *n* (%)**	47.2 (57.33)	11.8 (36.70)	**0.005**
**Anemia, *n* (%)**	22 (15.4)	39 (12.8)	0.463
**ECG QTc > 500 ms**	5 (3.5)	7 (2.3)	0.466
**Anticancer therapy, *n***			
Anthracycline Alkylating agents Fluoropyrimidines Antimetabolites VEGF Anti-HER2 therapy Hormonotherapy Proteasome inhibitors Monoclonal antibodies Multikinase inhibitors ICI	11 (7.7) 23 (16.1) 25 (17.5) 14 (9.8) 21 (14.7) 4 (2.8) 7 (4.9) 1 (0.7) 4 (2.8) 5 (3.5) 1 (0.7)	64 (21.1) 61 (20.1) 41 (13.5) 43 (14.1) 21 (6.9) 33 (10.9) 12 (3.9) 11 (3.6) 10 (3.3) 19 (6.1) 0	**<0.001** 0.315 0.267 0.198 **0.009** **0.004** 0.643 0.075 0.780 0.420 0.144
**Sinus rhythm, *n* (%)**	125 (87.4)	271 (89.1)	0.698
**Previous cancer, *n* (%)**	18 (12.6)	13 (4.3)	**0.001**
**Previous chemotherapy, *n* (%)**	17 (11.9)	29 (9.5)	0.446
**Previous radiotherapy, *n* (%)**	15 (10.5)	26 (8.6)	0.508
**Cancer progression, *n* (%)**	48 (33.6)	40 (13.2)	**<0.001**

* We grouped rare cancer types into “others” (skin, brain, pharyngeal, sarcomas). CAD—coronary artery disease; CRP—C-reactive protein; ECG—electrocardiogram; GFR—glomerular filtration rate; GLS—global longitudinal strain; ICI—immune checkpoint inhibitors; NP—natriuretic peptide; SD—standard deviation; VEGF—vascular endothelial growth factor.

**Table 3:** Univariate Cox regression analysis.

Factor	Category	HR (95% CI)	*p*-Value
**Age**		1.026 (1.011–1.041)	**<0.001**
**Male sex**	Yes	1.295 (0.922–1.820)	0.136
**Myocardial damage markers** Troponin elevation NP elevation Left ventricular systolic dysfunction Left ventricular diastolic dysfunction Abnormal GLS	Yes Yes Yes Yes Yes	0.937 (0.493–1.779) 2.030 (1.408–2.927) 1.478 (0.851–2.566) 2.264 (1.538–3.332) 2.665 (1.363–5.211)	0.842 **<0.001** 0.165 **<0.001** **0.004**
**Cancer location** Breast Gastrointestinal Hematologic Genitourinary Gynecologic Lung Other	Yes Yes Yes Yes Yes Yes Yes	0.267 (0.169–0.421) 1.273 (0.869–1.865) 0.694 (0.399–1.207) 1.600 (1.007–2.543) 2.052 (1.291–3.262) 4.142 (2.645–6.487) 1.370 (0.640–2.931)	**<0.001** 0.216 0.196 **0.047** **0.002** **<0.001** 0.417
**Cancer stage** II III IV	Yes Yes Yes	0.842 (0.292–2.425) 2.843 (1.107–7.304) 4.127 (1.641–10.381)	0.749 **0.030** **0.003**
**Metastatic cancer**	Yes	3.482 (2.499–4.851)	**<0.001**
**NYHA stage** I	Yes	1.762 (0.881–3.524)	0.109
II III IV	Yes Yes Yes	2.588 (1.630–4.110) 3.664 (2.102–6.388) 5.808 (0.783–43.090)	**<0.001** **<0.001** 0.085
**CV risk factors, *n* (%)** Hypertension Diabetes Dyslipidemia Smoking Obesity Prior CAD Valvular Heart Disease Kidney dysfunction (GFR < 60 mL/min/1.73 m^2^)	Yes Yes Yes Yes Yes Yes Yes Yes	0.949 (0.676–1.331) 0.873 (0.483–1.577) 0.447 (0.298–0.669) 0.547 (0.165–1.814) 0.912 (0.631–1.319) 1.016 (0.594–1.735) 1.500 (0.700–3.212) 2.013 (1.330–3.048)	0.760 0.652 **<0.001** 0.324 0.625 0.955 0.297 **<0.001**
**CRP elevation, *n* (%)**	Yes	1.007 (1.002–1.011)	**0.002**
**Anemia, *n* (%)**	Yes	1.929 (1.221–3.049)	**0.005**
**ECG QTc > 500 ms**	Yes	1.296 (0.530–3.169)	0.570
**Sinus rhythm, *n* (%)**	Yes	0.522 (0.129–2.114)	0.363
**Previous cancer, *n* (%)**	Yes	2.096 (1.278–3.438)	**0.003**
**Previous chemotherapy, *n* (%)**	Yes	1.301 (0.783–2.162)	0.310
**Previous radiotherapy, *n* (%)**	Yes	1.333 (0.780–2.279)	0.294
**Cancer progression**	Yes	3.118 (2.191–4.439)	**<0.001**

CAD—coronary artery disease; CRP—C-reactive protein; ECG—electrocardiogram; GFR—glomerular filtration rate; GLS—global longitudinal strain; NP—natriuretic peptide.

**Table 4:** Multivariate Cox regression analysis.

Factor	Category	HR (95% CI)	*p*-Value
**Age**		1.020 (1.005–1.036)	0.009
**Myocardial damage markers** Left ventricular diastolic dysfunction	Yes	1.731 (1.115–2.689)	0.015
**Cancer location** Breast Lung	Yes Yes	0.387 (0.241–0.621) 2.907 (1.826–4.627)	<0.001 <0.001
**Metastatic cancer**	Yes	2.208 (1.482–3.289)	<0.001
**NYHA stage** II III	Yes Yes	2.016 (1.242–3.272) 3.545 (1.948–6.450)	0.005 <0.001
**CV risk factors, *n* (%)** Dyslipidemia Kidney dysfunction (GFR < 60 mL/min/1.73 m^2^)	Yes Yes	0.438 (0.292–0.657) 2.085 (1.377–3.159)	<0.001 0.001
**Previous cancer, *n* (%)**	Yes	2.004 (1.219–3.295)	0.006
**Cancer progression**	Yes	1.853 (1.217–2.823)	0.004

GFR—glomerular filtration rate.

## Data Availability

All data and materials are available upon request.

## Abbreviations

ACEI	angiotensin-converting enzyme inhibitor
ARB	angiotensin receptor blocker
AH	arterial hypertension
BB	beta-blocker
BNP	brain natriuretic peptide
CAD	coronary artery disease
CCB	calcium channel blocker
Chemo	chemotherapy
CO	cardio-oncology
CRP	C-reactive protein
CTX	cardiotoxicity
CV	cardiovascular
CVRF	cardiovascular risk factor
DOAC	direct oral anticoagulant
DM	diabetes mellitus
DNA	deoxyribonucleic acid
GFR	glomerular filtration rate
GI	gastrointestinal
GLS	global longitudinal strain
GU	genitourinary
Gyn	gynecological
Hem	hematological
HF	heart failure
HFpEF	heart failure with preserved ejection fraction
Hgb	hemoglobin
HR	hazard ratio
ICI	immune checkpoint inhibitors
LMWH	low molecular weight heparin
LVEF	left ventricular ejection fraction
MRA	mineralocorticoid receptor antagonist
NP	natriuretic peptide
NT-proBNP	N-terminal pro-brain natriuretic peptide
PI	proteasome inhibitors
RES	reticuloendothelial system
RF	risk factor
RT	radiotherapy
S’	tricuspid annular systolic velocity by tissue Doppler
SAS	Statistical Analysis System
SD	standard deviation
TAPS	tricuspid annular plane systolic excursion
TKI	tyrosine kinase inhibitor
Tn	troponin;
Tn I	troponin I
VEGF	vascular endothelial growth factor
Vs	versus
VTE	venous thromboembolism
